# Clinical validation of ring‐mounted halcyon linac for lung SBRT: comparison to SBRT‐dedicated C‐arm linac treatments

**DOI:** 10.1002/acm2.13146

**Published:** 2020-12-20

**Authors:** Damodar Pokhrel, Justin Visak, Lana C. Critchfield, Joseph Stephen, Mark E. Bernard, Marcus Randall, Mahesh Kudrimoti

**Affiliations:** ^1^ Medical Physics Graduate Program Department of Radiation Medicine University of Kentucky Lexington KY USA

**Keywords:** AcurosXB Algorithm, Co/Nonplanar Geometry, FFF‐beam, lung SBRT, ring‐mounted Halcyon, VMAT

## Abstract

Stereotactic body radiotherapy (SBRT) of lung tumors via the ring‐mounted Halcyon Linac, a fast kilovoltage cone beam CT‐guided treatment with coplanar geometry, a single energy 6MV flattening filter free (FFF) beam and volumetric modulated arc therapy (VMAT) is a fast, safe, and feasible treatment modality for selected lung cancer patients. Four‐dimensional (4D) CT‐based treatment plans were generated using advanced AcurosXB algorithm with heterogeneity corrections using an SBRT board and Halcyon couch insert. Halcyon VMAT‐SBRT plans with stacked and staggered multileaf collimators produced highly conformal radiosurgical dose distribution to the target, lower intermediate dose spillage, and similar dose to adjacent organs at risks (OARs) compared to SBRT‐dedicated highly conformal clinical noncoplanar Truebeam VMAT plans following the RTOG‐0813 requirements. Due to low monitor units per fraction and less multileaf collimator (MLC) modulation, the Halcyon VMAT plan can deliver lung SBRT fractions with an overall treatment time of less than 15 min (for 50 Gy in five fractions), significantly improving patient comfort and clinic workflow. Higher pass rates of quality assurance results demonstrate a more accurate treatment delivery on Halcyon. We have implemented Halcyon for lung SBRT treatment in our clinic. We suggest others use Halcyon for lung SBRT treatments using abdominal compression or 4D CT‐based treatment planning, thus expanding the access of curative ultra‐hypofractionated treatments to other centers with only a Halcyon Linac. Clinical follow‐up results for patients treated on Halcyon Linac with lung SBRT is ongoing.

## 1 INTRODUCTION

Stereotactic body radiation therapy (SBRT) is an established treatment modality in the management of early‐stage nonsmall cell lung cancer (NSCLC) patients and provides a high cure rate 97% (median, 3 years actuarial) with minimal treatment‐related toxicity.^1^, ^2^, ^3^, ^4^, ^5^ Historically, SBRT has been delivered using 7–13 co/noncoplanar static beams or with dynamic conformal arcs (DCA).^6^, ^7^, ^8^, ^9^, ^10^ Lung SBRT can be delivered with intensity‐modulated radiation therapy (IMRT), robotic CyberKnife, or a Tomotherapy unit.^6^, ^7^, ^8^, ^9^, ^10^ For centrally located NSCLC, the Radiation Therapy Oncology Group (RTOG) 0813 trial provides recommendations for the dosing, contouring, treatment planning, and delivery of SBRT.^11^ This protocol is commonly used as a risk‐adapted prescription for tumors located adjacent to critical structures such as ribs. Traditional lung SBRT techniques are associated with long treatment times and patient inconvenience.^6^, ^7^, ^8^, ^9^, ^10^ Currently, lung SBRT is delivered via volumetric modulated arc therapy (VMAT).^12^, ^13^, ^14^ VMAT provides a more conformal dose distribution with faster dose fall‐off outside the target, better sparing of organs at risk (OAR) and quicker treatment delivery. The dosimetric advantages of VMAT for lung SBRT are enhanced by using a flattening filter free (FFF) beam instead of a traditional flattened beam.^15^, ^16^ These include higher dose rates, reduction of out‐of‐field dose, less head scatter, and less electron contamination.^15^, ^16^ These advantages directly improve the patient's treatments by improving target dose coverage at the lung‐tumor interface and shortening the treatment time.^13^, ^14^ Treatment time reduction potentially leads to improved patient comfort and decreased intrafraction target motion.

Varian recently introduced a single energy ring‐mounted linear accelerator, the Halcyon Linac (V2.0), for image‐guided radiation therapy.^17^ The compact Halcyon Linac is equipped with 6MV FFF beam and is capable of rotating the gantry at a speed of four rotations per minute.^18^, ^19^ The mean energy and the nominal depth of maximal dose are 1.3 MeV and 1.3 cm, compared to the Truebeam Linac (6MV FFF beam) at 1.4 MeV and 1.5 cm, respectively. Differing from the Truebeam Linac, the Halcyon is equipped with a new double stacked and staggered multileaf collimator (MLC) design. The upper and lower MLCs are offset by 5 mm allowing for a projected 5 mm effective width at isocenter. This produces a 5 mm modulation resolution similar to the Truebeam Linac. The Halcyon Linac allows for a maximum field size of 28 × 28 cm^2^ and unlike a traditional C‐arm Linac it does not have jaws. The MLC leaves on Halcyon are twice as fast as the standard millennium MLC, and the stacked design allow for ultralow leakage and transmission of <0.5%.^20^, ^21^Halcyon has less penumbra with a smaller dosimetric leaf gap (DLG) of 0.1 mm compared to traditional C‐arm Linacs. The Halcyon Linac additionally is equipped with a fast 15‐s kilovoltage cone beam CT (kV‐CBCT) imaging system that includes a high‐quality iterative CBCT reconstruction algorithm (iCBCT).^22^, ^23^ Daily patient setup times are significantly reduced on the Halcyon with a new one‐step setup that will automatically apply couch shifts during a patient's setup. Thus, eliminating the need to manually apply isocenter shifts.

Some investigators have shown fast and effective treatment delivery is possible with Halcyon for conventionally fractionated breast, head and neck, and prostate treatments.^24^, ^25^, ^26^ For hypofractionation schemes, Knutson et al. reported a retrospective dosimetric study of fractionated intracranial stereotactic radiation therapy (SRT) using the Halcyon.^27^ In a 20‐patient study (30 Gy in five fractions), they demonstrated acceptable plan quality for brain SRT using Halcyon coplanar geometry. Another study by Li et al. demonstrated the Halcyon V2.0 can generate plan quality comparable to a C‐arm Linac for 6–10 brain tumors (diameter >1.0 cm), with a single‐isocenter VMAT approach for intracranial radiosurgery.^28^ While these retrospective planning studies demonstrated acceptable plan quality, they failed to include setup uncertainties associated with plan delivery and did not use their plans for patient treatment. Our study is the first to focus on the evaluation and clinical implementation of Halcyon Linac for lung SBRT. In this report, we have evaluated plan quality, treatment delivery efficiency, and accuracy of SBRT for lung tumors using Halcyon Linac by comparing with high‐quality noncoplanar clinical VMAT plans on our SBRT‐dedicated Truebeam Linac. This study provides the support for lung SBRT treatments with Halcyon. Lung SBRT has been clinically implemented in our clinic based on these findings.

## 2 MATERIALS AND METHODS

### 2.1 Halcyon Linac for SBRT

After installation of a Halcyon V2.0, it was confirmed the machine met manufacturer specifications through both initial acceptance testing and commissioning. These high‐quality lung SBRT plans are created with Eclipse using the advanced AcurosXB algorithm^29^, ^30^, ^31^, ^32^ as the final dose calculation as it has been shown it better predicts dose distribution by accounting for tissues heterogeneities (e.g., the SBRT board and Halcyon couch insert). Independent validation was performed using the MD Anderson's SBRT credentialing service by delivering a SBRT prescription dose of 6.6 Gy to the provided phantom. All dosimetric criteria established by IROC for SBRT treatments were satisfied. Currently, tumor motion is managed via abdominal compression and 4D CT‐based target delineation or both as needed.

### 2.2 Patient characteristics

After Institutional Review Board approval, 15 consecutive early stage I‐II NSCLC patients with centrally located tumors who underwent lung SBRT treatments using highly conformal noncoplanar clinical VMAT plans on a SBRT‐dedicated Truebeam Linac for 50 Gy in five fractions were included in this retrospective study. Per institutional protocol, our physicians predominately treat lung SBRT patients using a noncoplanar geometry to ensure maximal target conformality and OAR sparing.

### 2.3 Imaging and target definition

Patients were immobilized using the Body Pro‐Lok^TM^ platform (CIVCO system, Orange City, IA, USA) in the supine position with arms up above their head with an abdominal compression possibly decreasing diaphragmatic motion to less than 1.0 cm. A free‐breathing planning 3D CT scan was acquired on a GE Lightspeed 16 slice CT scanner (General Electric Medical Systems, Waukesha, WI, USA) with 512 × 512 pixels at 2.5 mm slice thickness in the axial helical mode. Following the 3D CT scan these patients underwent a respiration‐correlated limited 4D CT scan (about 2 cm above and 2 cm below the tumor extend seen on 3D CT scan) using the Varian RPM System (version 1.7) in the same position. Our limited 4D CT images were reconstructed in ten equally spaced phase bins using an Advantage 4D Workstation (GE Medical Systems, San Francisco, CA, USA), where the maximum intensity projection (MIP) images were generated. The planning 3D CT and the MIP images were imported into Eclipse TPS (Version 15.6, Varian Medical Systems, Palo Alto, CA, USA) and coregistered for tumor delineation. An internal target volume (ITV) was created using the 4D‐MIP and the planning target volume (PTV) was generated by adding a 0.5 cm symmetric margin around the ITV per RTOG‐0813 recommendation.^11^ The relevant critical structures that were contoured included bilateral lungs excluding the PTV (normal lung), spinal cord, heart, trachea/bronchus, esophagus, ribs, and skin.

### 2.4 Clinical Truebeam VMAT plans

Our clinical highly conformal VMAT lung SBRT plans were generated in Eclipse TPS using 3–6 (mean, 4) partial noncoplanar arcs (with ±5–10° couch kicks) on a SBRT‐dedicated Truebeam Linac (Varian Medical Systems, Palo Alto, CA, USA) equipped with a standard millennium 120 MLC and a 6MV FFF (1400 MU/min) beam per our departmental SBRT protocol. The SBRT board was inserted into the plan. The isocenter position was set to the geometric center of the PTV. These partial noncoplanar arcs had an arc length of approximately 180‐200°, and collimator angles (between 30° and 135°) were manually optimized to reduce MLC tongue‐and‐groove leakage dose throughout the arc rotation. Jaw‐tracking option was enabled during plan optimization to further minimize out‐of‐field dose. Dose was prescribed to the 70‐80% isodose line and normalized to ensure at least 95% of the PTV received the prescribed dose. No hot spots were allowed outside of the PTV. All clinical treatment plans were calculated with the advanced AcurosXB (Varian Eclipse TPS, Version 15.6) dose calculation algorithm^29^, ^30^, ^31^, ^32^ on the planning 3D CT images with heterogeneity corrections, 1.25 × 1.25 × 1.25 mm^3^ calculation grid size (CGS), and the Photon Optimizer (PO) MLC algorithm. Dose to medium reporting mode was applied. Planning objectives followed the RTOG‐0813 requirements for prescription isodose surface coverage, target dose heterogeneity, high and low dose spillages, and dose limiting organ constraints.^11^ These patients were treated on Truebeam Linac every other day per lung SBRT protocol.

### 2.5 Halcyon VMAT plans

For comparison, all clinical plans were reoptimized in Eclipse TPS using the same numbers of partial arcs, identical collimator rotations and identical arc geometry (including Truebeam VMAT arc length). All Halcyon arcs were coplanar due to geometric limitations. Additionally, the Halcyon couch and SBRT board was inserted. Optimization objectives were identical to Truebeam VMAT plans. Identical dose calculation algorithm, dose reporting mode, CGS and PO MLC optimizer were used for Halcyon plans as described above. Halcyon VMAT plans were normalized identically to clinical Truebeam VMAT plans as described above, and the ITV hot spots were limited to those of the respective Truebeam VMAT plans.

### 2.6 Plan comparison and statistical analysis

The clinical Truebeam VMAT and Halcyon VMAT plans were compared via RTOG‐0813 SBRT protocol for target dose conformity (CI), tumor dose heterogeneity (HI), gradient index (GI), and dose to OAR. Additionally, delivery efficiency and accuracy were recorded. The DVHs of all treatment plans were evaluated following RTOG‐0813 high and intermediate dose spillage parameters as follows^11^:

1. Conformity index, CI: ratio of prescription isodose volume to the PTV. CI less than 1.2 is desirable; CI = 1.2–1.5 is acceptable with minor deviations.
2. Gradient index, GI: ratio of 50% prescription isodose volume to the PTV. GI must be smaller than 3–6, depending on the PTV.
3. Maximum dose at any point 2 cm away from the PTV margin in any direction, D_2cm_: D_2cm_ must be smaller than 50–70%, depending on the PTV size.
4. Percentage of normal lung receiving dose equal to 20 Gy or more, V20Gy: Per protocol, V20Gy should be less than 10%, V20Gy less than 15% is acceptable with minor deviations.
5. Heterogeneity index, HI: HI = Dmax/prescribed dose was used to evaluate the dose heterogeneity within the PTV.
6. Gradient distance, GD: GD is the average distance from 100% prescribed dose to 50% prescribed dose, which indicates how sharply the dose falls off. The GD is used to evaluate dose sparing to normal lung volume. The smaller the GD, the faster the dose fall‐off around the target.
7. Total number of monitor units (MU) per fraction.
8. Modulation factor, MF: ratio of total number of MU per fraction to the prescription dose in cGy.
9. Beam‐on time, BOT: BOT was recorded during portal dosimetry QA measurement at the machine.

All clinical Truebeam VMAT and Halcyon VMAT plans were evaluated for the volume of normal lung receiving 10 Gy, 5 Gy and mean lung dose (MLD), dose to the spinal cord (maximum and 0.25 cc), heart (maximum and 15 cc), trachea/bronchial tree (maximum and 4 cc) and esophagus (maximum and 5 cc). Doses to ribs (maximum and 1 cc) and skin (maximum and 10 cc). The mean and standard deviation for each dose metric was compared using a two‐tailed paired Student's t‐test (using an upper bound *P*‐value of <0.05, being statistically significant) for all dosimetric parameters, target coverage, dose limits to OAR and treatment delivery parameters. Maximal dose limits to organs are as follows: spinal cord <30.0 Gy (0.25 cc < 22.5 Gy), heart <52.5 Gy (15 cc < 32.0 Gy), esophagus <52.5 Gy (5 cc < 27.5 Gy), trachea/bronchial tree <52.5 Gy (4 cc < 18 Gy), and skin <32.0 Gy (10 cc < 30 Gy). Dosimetric verification of both plans were performed using a portal dosimetry (PD) quality assurance (QA) procedure established in our clinic.^33^, ^34^ Our institution performs patient‐specific QA using the electronic portal imaging device (EPID, aS1200 flat panel detector, Varian Medical Systems) mounted on the Truebeam and Halcyon Linacs. This detector has an active area of 400 mm × 400 mm with a high‐resolution pixel size of 0.34 mm making it an excellent device for the QA of lung SBRT plans.

## 3 RESULTS

### 3.1 Target coverage and intermediate dose spills

The average ITV was 13.0 ± 15.1 cc (range, 1.5–61.2 cc). The mean PTV was 40.9 ± 29.2 cc (range, 9.7–114.3 cc). This corresponds to an average tumor diameter of 3.9 ± 1.0 cm (range, 2.6–5.9 cm). Table 1 summarizes the high and intermediate dose spillage. Both plans were acceptable per RTOG‐0813 requirements. Compared to noncoplanar SBRT‐dedicated Truebeam VMAT plans, coplanar Halcyon VMAT plans showed similar tumor conformity, tumor dose heterogeneity and similar GTV doses (minimum, maximal and mean) with no statistically significance differences. Near minimum dose to PTV (PTVD99) was similar between the plans and met protocol requirement. Even with coplanar geometry restrictions, Halcyon VMAT plans show similar gradient indices and similar intermediate dose‐spillage (GI, D_2cm_ and GD, see Table 1). Although these values are slightly higher, they are still acceptable per SBRT protocol. Halcyon's GI (*P* = 0.004) and GD (*P* = 0.001) small absolute differences may not be clinically significant.

**TABLE 1:** Evaluation of target coverage for 15 lung SBRT patients for both plans. Prescription was 50 Gy in five fractions.

Target volume	Parameters	Truebeam VMAT	Halcyon VMAT	*P*‐value
PTV	PTVD99 (Gy)	49.1 ± 1.9 (47.8–56.2)	48.7 ± 1.5 (47.7–54.1)	**0.04**
	CI	1.00 ± 0.03 (0.97–1.07)	1.01 ± 0.03 (0.98–1.09)	n. s.
	HI	1.23 ± 0.04 (1.2–1.4)	1.24 ± 0.03 (1.2–1.3)	n. s.
	GI	4.34 ± 0.9 (3.40–7.07)	4.64 ± 1.1 (3.5–7.6)	**0.004**
	D_2cm_ (%)	51.7 ± 4.5 (44.6–61.6)	52.1 ± 5.5 (44.9–62.3)	n. s.
	GD (cm)	1.16 ± 0.23 (0.83–1.62)	1.23 ± 0.25 (0.85–1.71)	**0.001**
ITV	D_min_ (Gy)	53.4 ± 2.3 (49.4–56.9)	53.4 ± 2.5 (48.9–58.2)	n. s.
	D_max_ (Gy)	61.5 ± 2.5 (57.7–68.3)	62.2 ± 1.7 (59.0–65.7)	n. s.
	D_mean_ (Gy)	58.1 ± 1.9 (55.4–63.1)	58.6 ± 1.71 (55.5–61.8)	n. s.

Mean ± SD (range) was reported. n. s., not statistically significant.

### 3.2 Dose to OAR

The dosimetric differences (mean, standard deviation and range) between clinical Truebeam VMAT and Halcyon VMAT plans for the OAR (spinal cord, heart, esophagus, trachea/bronchus, skin, ribs and normal lung) are listed in Table 2. No major or clinically significant differences were observed. Both plans achieved RTOG‐0813 protocol compliance criteria. Statistically insignificant differences were found for most of the evaluated dosimetric parameters excluding dose to 3 cc of esophagus, 10 cc of skin, normal lung V10Gy, V5Gy, and MLD (see, highlighted *P*‐values <0.05). Despite the reported statistical significance, absolute differences were about 1.0 Gy, 1.0 Gy, 0.4%, 0.9%, and 0.2 Gy, respectively. This suggests that these small differences are not clinically significant given they are well below RTOG protocol guidelines.

**TABLE 2:** Evaluation of dose to OAR for 15 lung SBRT patients for both plans. Prescription was 50 Gy in five fractions.

Dose to OAR	Parameters	Truebeam VMAT	Halcyon VMAT	*P*‐value
Spinal cord (Gy)	D_max_	8.3 ± 5.0 (1.7–16.1)	8.8 ± 4.1 (3.1–14.9)	n. s.
	D_0.25cc_	7.5 ± 4.5 (1.5–13.8)	8.0 ± 3.7 (2.8–13.5)	n. s.
Heart/pericardium (Gy)	D_max_	22.2 ± 17.4 (0.31–53.6)	22.6 ± 17.7 (0.39–53.6)	n. s.
	D_15cc_	10.5 ± 8.5 (0.2–33.2)	10.8 ± 8.6 (0.27–34.1)	n. s.
Esophagus (Gy)	D_max_	12.0 ± 9.4 (2.5–41.0)	12.7 ± 8.8 (3.8–40.5)	n. s.
	D_3cc_	7.6 ± 5.6 (1.9–24.7)	8.5 ± 5.7 (2.8–26.2)	**0.004**
Trachea/bronchial tree (Gy)	D_max_	17.8 ± 19.0 (0.3–55.4)	17.3 ± 18.7 (0.4–54.5)	n. s.
	D_4cc_	8.1 ± 10.4 (0.2–36.6)	8.2 ± 10.5 (0.3–37.3)	n. s.
Skin (Gy)	D_max_	17.1 ± 5.1 (8.8–28.3)	17.7 ± 4.9 (10.6–28.0)	n. s.
	D_10cc_	10.7 ± 3.1 (6.1–16.5)	11.6 ± 3.0 (7.5–16.9)	**0.001**
Ribs (Gy)	D_max_	45.6 ± 10.1 (28.8–57.2)	46.0 ± 10.6 (27.8–58.0)	n. s.
	D_1cc_	37.1 ± 9.6 (24.9–53.1)	37.1 ± 9.5 (23.9–53.6)	n. s.
Normal lung	V_20Gy_ (%)	3.3 ± 2.3 (0.7–9.7)	3.5 ± 2.5 (0.7–10.0)	n. s.
	V_10Gy_ (%)	8.0 ± 4.5 (2.3–20.7)	8.4 ± 4.5 (2.6–21.0)	**<0.001**
	V_5Gy_ (%)	13.7 ± 5.7 (6.2–28.4)	14.6 ± 5.8 (7.0–29.1)	**0.001**
	MLD (Gy)	2.8 ± 1.3 (1.2–6.3)	3.0 ± 1.3 (1.4–6.7)	**<0.001**

Mean ± SD (range) was reported. n. s. = not statistically significant.

**FIG. 1:**
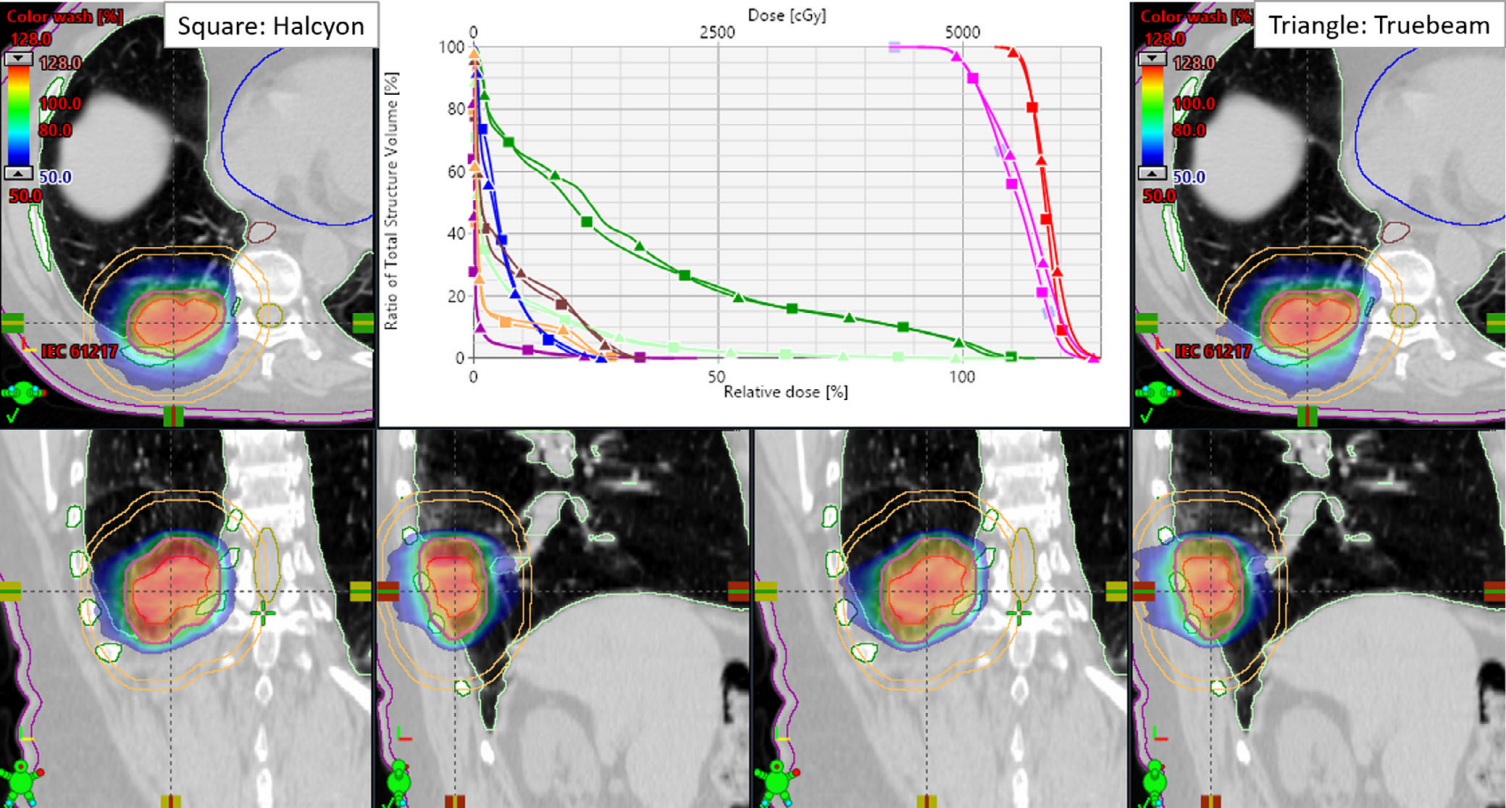
Comparison of Halcyon VMAT vs Truebeam VMAT plan for patient #10. The isodose colorwash for the Halcyon VMAT (left panel) vs clinical Truebeam VMAT plan (right panel) is shown, the crosshair showing the isocenter location. Critical structures shown are ribs (green), spinal cord (yellow), normal lung (light green), heart (blue), esophagus (brown), skin (magenta) as well as D2cm ring (peach). The top middle panel shows the DVH comparison for both plans with identical PTV coverage (pink) and similar dose to the GTV (red). Triangles are Truebeam VMAT and squares are the Halcyon VMAT. Identical target coverage and similar OAR sparing were achieved on both plans.

Figure 1 shows the SBRT dose distribution in the axial, coronal, and sagittal views through the isocenter plane (cross hair) for an example patient (#10) planned with Truebeam VMAT (right panel) and Halcyon VMAT (left panel). Even with the coplanar arc geometry, Halcyon VMAT produced a similar or tighter 50% isodose distribution (see blue isodose lines) compared to the Truebeam VMAT plan. The DVH parameters (top middle panel) compared for target coverage and dose to OAR suggest dosimetrically comparable plans. The PTV size was 59.0 cc (4.8 cm diameter). This tumor size was relatively large for the patient cohort and was in the right lower lobe. The CI, HI, GI, D_2cm_, GD, and normal lung V_20Gy_ were 1.02 vs. 1.01, 1.25 vs. 1.28, 3.40 vs. 3.38, 50.1% vs. 53.4%, 1.25 cm vs. 1.24 cm, and 3.7% vs. 3.6% for Halcyon VMAT and clinical Truebeam VMAT plans, respectively. All dosimetric parameters (including dose to OAR) were similar between the plans and within the RTOG‐0813 compliance criteria.

### 3.3 Treatment delivery efficiency and accuracy

Compared to SBRT‐dedicated Truebeam VMAT plans, Halcyon VMAT plans delivered lower total MU indicating less beam modulation. Mean values of total MU (*P* < 0.001) and MF (*P* < 0.001) were 3450 and 3.45 for Truebeam VMAT plans vs 3128 and 3.13 for Halcyon (Table 3). Less MLC modulation is desirable for lung SBRT plans favoring Halcyon VMAT. The beam‐on time and the PD QA pass rates for Truebeam VMAT vs Halcyon VMAT plans are shown in Table 3. Despite the less total MU and small MF, the mean total beam on time at Halcyon Linac plans (3.9 min, up to 6.3 min) was longer with respect to Truebeam VMAT plans (2.6 min, up to 4.3 min) (*P* < 0.001). The Halcyon is at a disadvantage as the maximal achievable dose rate at Halcyon was 800 MU/min vs 1400 MU/min for the Truebeam. Despite this increase of beam‐on time, cumulative treatment time could be similar or even shorter on Halcyon because of the previously explained one‐step setup capability. Quicker setups will lower the total time a patient is on the table, potentially reducing errors due to intrafraction tumor motion.

**TABLE 3:** Comparison of average values of treatment delivery parameters (and range) between Truebeam VMAT and replanned Halcyon VMAT plans for 15 lung SBRT patients.

Beam delivery parameters	Truebeam VMAT	Halcyon VMAT	*p*‐value
Total monitor units (MU)	3450 ± 807 (2656–5945)	3128 ± 722 (1976–5139)	**0.034**
Modulation factor (MF)	3.45 ± 0.81 (2.66–5.95)	3.13 ± 0.72 (1.98–5.14)	**0.034**
Beam‐on time (min)	2.56 ± 0.58 (2.11–4.25)	3.91 ± 0.90 (2.47–6.32)	**<0.001**
γ‐pass rate (%) [2%/2mm]	93.0 ± 2.5 (91.4–96.5)	94.4 ± 2.1 (93.5–97.8)	**0.041**

Statistically significant values are highlighted in bold.

As mentioned above, treatment delivery accuracy of lung SBRT was evaluated by delivering each plan in QA mode to both Linac's on‐board EPID and recording the gamma analysis pass rate via portal dosimetry. The dose delivery accuracy of the Truebeam VMAT plans and the corresponding Halcyon VMAT plans were 93.0 ± 2.5% (ranged, 91.4–96.5%) and 94.4 ± 2.1% (ranged, 93.5–97.8%) on average, respectively. This pass rate was assessed with a 2%/2mm global gamma criteria with a low‐dose threshold of 10%. Halcyon VMAT plans show significantly better QA pass rates (*P* = 0.041) due to less beam modulation compared to those of Truebeam. Due to the small pixel size of aS1200 EPID detector (0.34 mm), the 3%/3mm clinical gamma criteria was not very useful to identify the dosimetric differences for these lung SBRT plans due to pixels averaging effect. Therefore our clinical practice is to use 2%/2mm gamma criteria for lung SBRT while using PD QA procedure.

### 3.4 First lung SBRT patient treated on Halcyon

#### 3.4.1 Plan quality

**FIG. 2:**
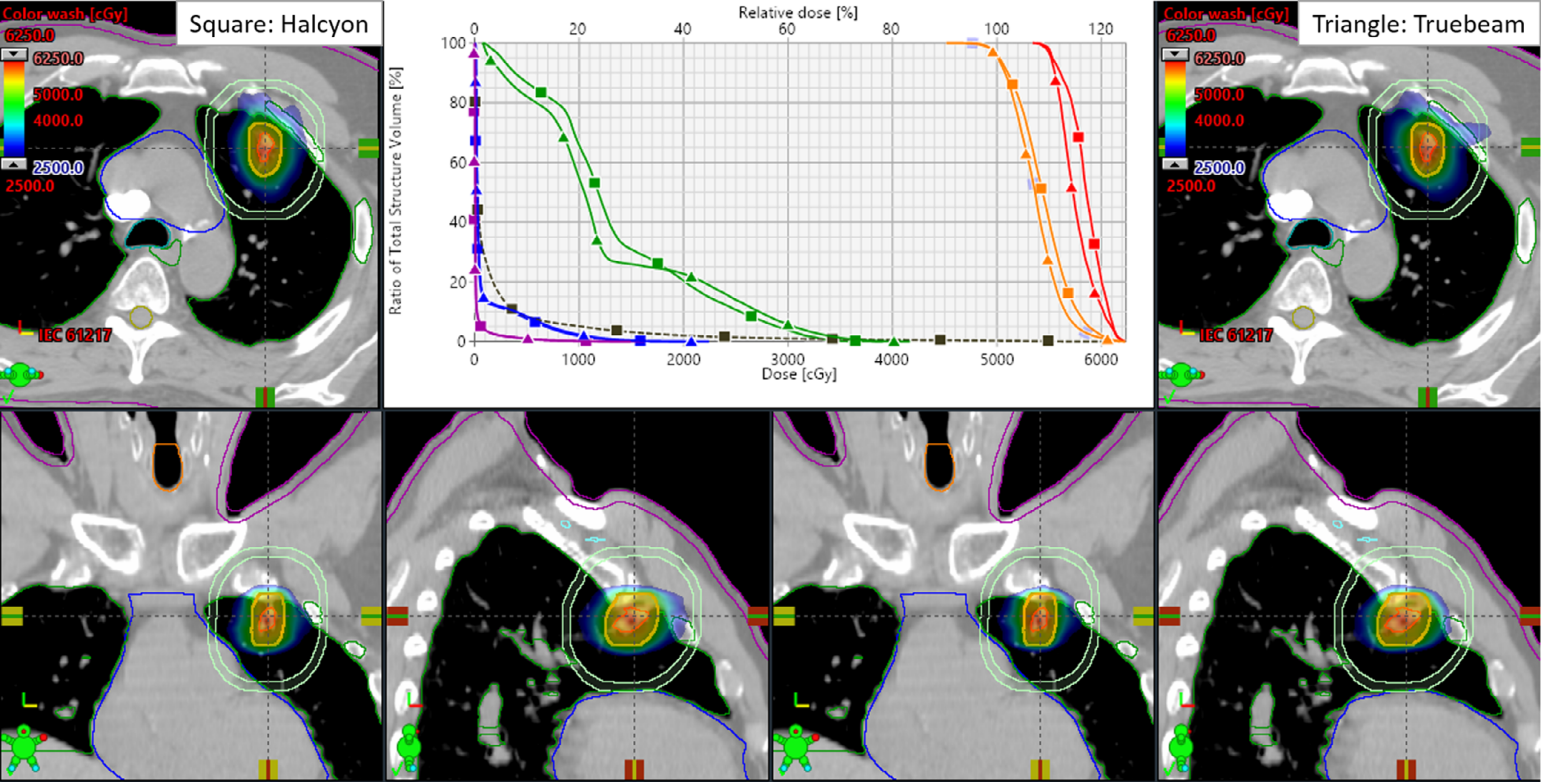
Comparison of coplanar Halcyon VMAT vs noncoplanar Truebeam VMAT plan for the first lung cancer treated with SBRT on Halcyon in our center. The isodose color wash for the Halcyon VMAT (left panel) vs Truebeam VMAT plan (right panel) is shown. Crosshair shows the isocenter location. Critical structures shown were ribs, cord, normal lung, heart, esophagus as well as D_2cm_ ring (sky blue). The top middle panel shows the DVH comparison for both plans. Triangles are Truebeam VMAT and squares are Halcyon VMAT (red, ITV; orange, PTV; green, ribs; brown, normal lung; blue, heart, and magenta, skin). Identical target coverage and similar OAR sparing were achieved on both plans.

Based on these results, we have implemented lung SBRT treatment on our Halcyon Linac. This is the first patient who underwent lung SBRT on our Halcyon Linac received 50 Gy in five treatments every other day for a left upper lobe lung lesion. The PTV size was 10.0 cc (2.7 cm diameter). Three coplanar partial arcs with an arc length 200° were used with three‐different collimator rotations. In this case, the CI, HI, GI, D_2cm,_ and normal lung V_20Gy_ were 1.03, 1.21, 4.8, 47.5%, and 2% and all were RTOG‐0813 compliant. The maximal dose to spinal cord, (<5.0 Gy), heart (<23.0 Gy), esophagus (<9.0 Gy), bronchial tree (<8.0 Gy), skin (<15.0 Gy), and ribs (<42.0 Gy) were well below the RTOG compliance criteria. Total MU per fraction was 3062. The MF and total beam‐on time was 3.06 and 3.83 min, respectively. The net treatment time (from first arc on until last arc off, including second and third arc preparation time, but no couch kick time) was about 4.0 min. For this patient, recorded mean couch time (including one‐step patient setup, 15‐s kV cone beam CT imaging and tumor matching) was less than 10 min. Figure 2 shows the SBRT dose distributions in 3 views through the isocenter for this patient treated with coplanar Halcyon VMAT (left panel) compared to noncoplanar VMAT plan (right panel) generated for comparison using identical three partial arcs (arc length, collimators setting) but with 0°, +10 ^o^, and −10° couch kicks on a SBRT‐dedicated Truebeam Linac. Halcyon Linac provided a clinically desirable tighter 50% isodose distribution (see blue isodose line) similar to Truebeam. This suggests dosimetrically comparable plan quality.

#### 3.4.2 Delivery efficiency and accuracy

**FIG. 3:**
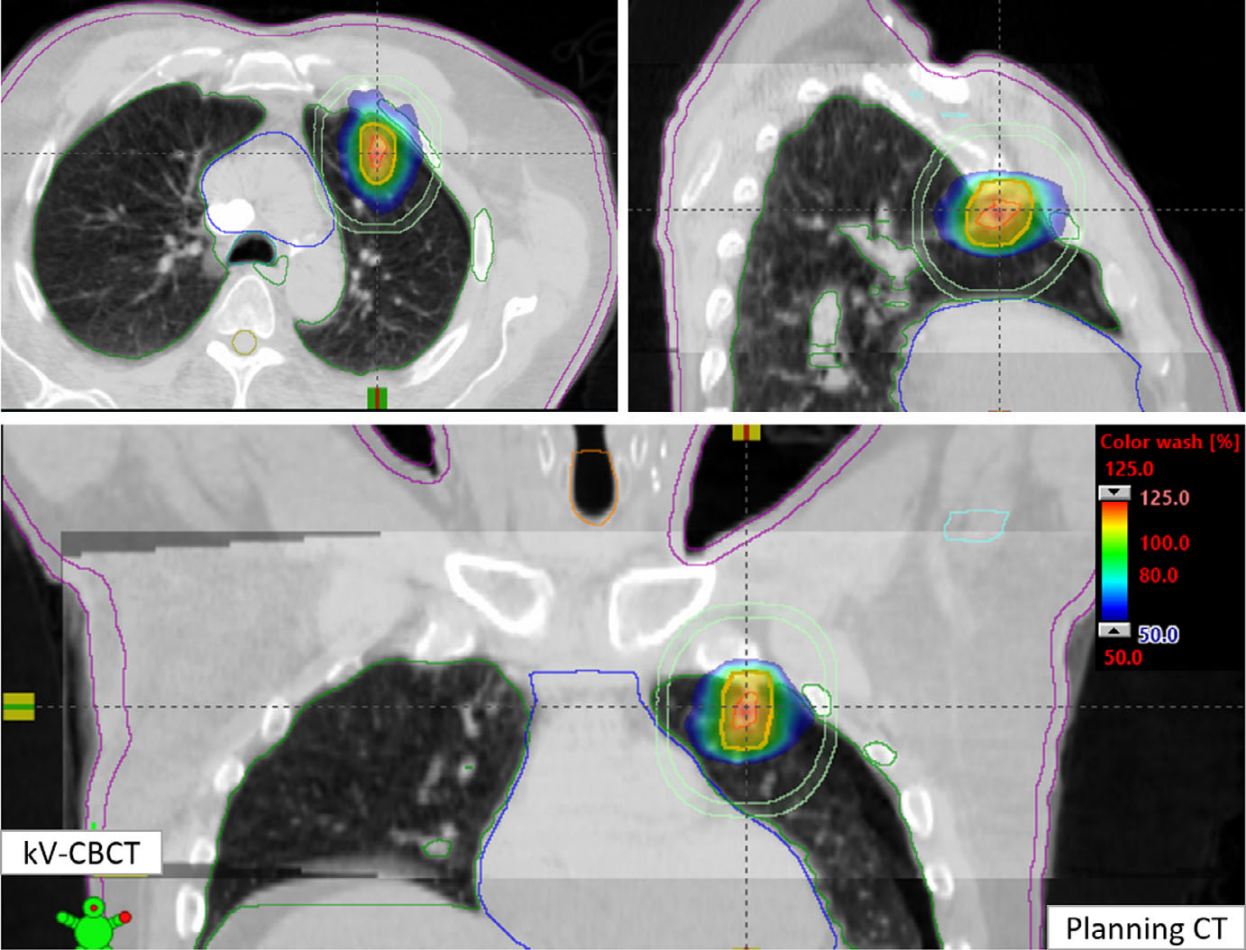
Axial, coronal and sagittal views of Halcyon kV‐CBCT images (see inset) coregistered with planning CT images (see back of coronal and sagittal views) used for image‐guided SBRT on Halcyon. In addition to anatomical landmarks, the planned dose cloud was superimposed for this treatment. Halcyon kV‐CBCT images were acquired in the treatment position in free breathing with abdominal compression. 3D soft‐tissue matching was performed via auto‐registration of online kV‐CBCT with the planning CT followed by manually fine‐tuning for setup corrections.

Pretreatment PD QA pass rate was 95.3% with a 2%/2mm gamma passing criteria. The net treatment time (from first beam on until last beam off, including second and third beam preparation, but no couch kick time) was about 4.0 min as described above. Recorded mean couch time for this patient on Halcyon Linac was less than 10 min. This patient was initially positioned using external marks and in‐room lasers, followed by the one‐step patient setup and a 15 s pretreatment free‐breathing kV‐CBCT scan. An in‐house SBRT/IGRT protocol was applied to coregister the pretreatment kV‐CBCT with the planning CT scans (see Fig. 3). Image registration was performed automatically based on region of interest and bony landmarks. Registration was followed by manual refinement and confirmed by the treating physician and physicist. The patient position was then corrected for three degrees of freedom (DOF) according to the results of soft tissue registration and the treatment was delivered. Those three‐DOF couch corrections were within the limits of departmental SBRT protocol guidelines for this patient (translational shifts within ±3.0 mm in each direction). The entire imaging and delivery sequences were monitored and verified by the treating physician and physicist. Figure 2 shows the planned isodose color wash superimposed with the daily Halcyon kV‐CBCT images after the translational couch corrections were applied.

## 4 DISCUSSION

We have evaluated the plan quality, treatment delivery efficiency, and accuracy of lung SBRT treatment using the Halcyon platform. All VMAT lung SBRT plans generated using coplanar Halcyon beam geometry had similar dosimetric plan quality compared to our standard SBRT‐dedicated clinical noncoplanar Truebeam VMAT plans including dose to ITV, target conformity, tumor dose heterogeneity, and gradient indices. Additionally, all Halcyon plans met RTOG‐0813 requirements and achieved similar target coverage (Table 1) compared to noncoplanar clinical VMAT plans. Halcyon VMAT plans provided similar or better OAR (spinal cord, heart, esophagus, trachea/bronchus, ribs, and skin, see Table 2) sparing and were well below protocol dose requirements. Halcyon VMAT plans required less MU to deliver the same dose due to less beam modulation across the target. Although, the beam on times were longer for Halcyon plans (about 2 min, on average) than Truebeam, this is mainly due to the maximum dose rate of 800 MU/min for the Halcyon Linac vs. 1400 MU/min for Truebeam with 6MV FFF beam. However, if the time that the therapists must enter at Truebeam to apply couch kicks is accounted for, the overall patient's treatment time could be similar or even less with Halcyon. Furthermore, the treatment delivery accuracy was improved significantly (Table 3) when 2%/2 mm gamma passing criteria was recorded. Based on these results, Halcyon for lung SBRT has been implemented in our center for selected lung SBRT patients. There could be a potential concern that the interplay effects between very high dynamic MLC modulation and tumor motion may further degrade delivery accuracy.^35^, ^36^ Even though this study does not quantify the variation of the delivered dose due to the tumor motion that need to be verified in the future for Halcyon Linac. Better delivery accuracy of Halcyon VMAT plans can arguably address the major concerns of small‐field dosimetry errors and MLC interplay effects that propagate in highly modulated Truebeam VMAT plans. Therefore, one benefit of treating lung SBRT on the Halcyon is the same prescription dose can be delivered with reduced total MU and MF.

For highly conformal lung SBRT, Dong *et al*.^37^compared 4π plans with seven to nine static‐beam IMRT plans and VMAT plans prescribed to 50 Gy in four fractions for 12 centrally located lung cancer patients. The 4π algorithm used up to 30 optimized coplanar/noncoplanar IMRT fields and it was concluded that, compared to IMRT or VMAT, the 4π plans gave significantly and consistently better target coverage and spared OAR to a greater degree. However, the total MU and treatment delivery time of the 4π treatment was not reported. It is likely that delivering 30 co/noncoplanar treatment fields to treat lung SBRT patients would be clinically unfeasible due to potential collision issues and the therapist's need to enter the treatment room many times. Utilizing Halcyon VMAT overcomes this concern. The O‐ring design of the Halcyon allows it to deliver SBRT treatments faster (within 15 min) without patient collision issues, compared to 4π treatment, robotic CyberKnife (~45 min) or Tomotherapy unit (30‐40 min).^6^, ^7^, ^8^, ^9^, ^38^, ^39^ By shortening the overall treatment delivery time, the risk of deviating from planned dose delivery is decreased as the patient is less likely to move on the treatment table (e.g., coughing, distress, or self‐adjusting due to pain) and cause a geometric miss.

Due to the coplanar geometry, other possible concerns for lung SBRT treatments on Halcyon could be low‐ and intermediate dose spills in the chest wall and ribs,^40^, ^41^ normal lung (V_20Gy_, V_10Gy_, and V_5Gy_)^42^, ^43^ and dose to skin.^44^, ^45^ For example, Pettersson *et al*.^40^ studied a large cohort of 68 NSCLC patients treated to 45 Gy in three fractions with lung SBRT using coplanar/noncoplanar beams. Of the 33 patients with a complete clinical and radiographic follow‐up exceeding 15 months, they reported 13 total rib fractures in seven patients. In their study, the logistic dose‐response curve related the risk of radiation‐induced rib injury to the dose to 2 cc of the rib. For a median follow‐up of 29 months, they showed that the 2 cc of rib receiving 27.3 Gy in three fractions had a 5% chance of rib fracture. In current study, the Halcyon VMAT plans provided a sharp dose fall‐off, limiting dose to the rib (less leakage and transmission) and other OAR compared to Truebeam. O'Grady *et al* have shown a slight increase in superficial skin dose in whole‐breast irradiation with Halcyon compared to traditional C‐arm Linac with flattened beam but presented the argument that additional superficial dose could help by limiting the need for bolus.^45^ While the current study showed the skin received slightly higher doses with Halcyon VMAT treatment, the average dose received by the skin was significantly below RTOG‐0813 compliance criteria and is not considered clinically significant. However, clinical follow‐up results including tumor‐control and treatment related toxicities in patients treated on Halcyon Linac is essential and is ongoing.

There are a few caveats in this study. First, Halcyon's maximal dose rate of 800 MU/min is significantly less than the Truebeam's noncoplanar VMAT maximum dose rate of 1400 MU/min for 6MV FFF beam. This difference leads to an increase of BOT in Halcyon VMAT plans, although overall treatment time would be similar as described above. Upgrading Halcyon's maximum achievable dose rates to a practical 1000 MU/min may potentially match or improve BOT relative to Truebeam. This may aid in improved treatment delivery efficiency for a 50–55 Gy in five fractions treatment scheme. However, delivering an ultrahigh single dose of 30 Gy or 34 Gy^46^, ^47^ or more drastic hypofractionated 54–60 Gy in three fractions treatment schemata^48^ on Halcyon Linac for lung SBRT may take longer treatment times for the patient on the table. Therefore, we currently only recommend treating selective lung SBRT patients with 50–55 Gy in five fractions on the Halcyon. On Halcyon, more studies are needed to investigate these ultrahigh dosing schemes. The second caveat is that the Truebeam with a 6‐DOF couch may better reduce the rotational setup errors in treatment delivery. However, as of now, the exact dosimetric impacts of 6‐DOF couch corrections for lung SBRT on Halcyon are not known. Another caveat is that the Halcyon Linac does not have a full package of motion management system available yet. As of now, fully automated deep inspiration breath hold (DIBH) or phase‐gated lung SBRT treatments are not possible on Halcyon. Therefore, currently we rely on abdominal compression or 4D CT‐based ITV planning for lung SBRT treatments on Halcyon Linac.

In summary, relatively faster or similar overall treatment delivery is possible with the Halcyon Linac and eliminates patient collision issues (therapists do not need to enter treatment room many times for couch rotations). This potentially benefits patients who cannot lie flat in the treatment position for an extended treatment time and may lower the risk of intrafraction motion error. Reducing beam modulation on Halcyon VMAT minimizes the major concerns over accuracy of dose calculation and delivery errors for small fields (beamlets) in areas of tissue/lung interfaces. This may also improve susceptibility to interplay effects. At the time of this report, we are actively treating select lung SBRT patients on Halcyon Linac to provide them a fast setup (i.e., shorter cumulative treatment time), superior imaging and overall improved quality of treatment. Clinical follow‐up for these lung SBRT patients is underway. The Halcyon Linac can be adapted to other disease sites such as fractionated stereotactic treatment of brain, abdominal/pelvis lesions such as liver, pancreas and adrenal glands and vertebral SBRT. Due to decreased MU/treatment and relatively smaller beam on time with Halcyon VMAT, DIBH to liver, and lung SBRT treatments merits future investigation. Quantification of dosimetric impacts of rotational corrections for lung SBRT treatments on Halcyon Linac is ongoing.

## 5 CONCLUSION

This report demonstrates the treatment planning feasibility, delivery efficiency and accuracy, and clinical implementation of lung SBRT on ring‐mounted Halcyon Linac for selected lung SBRT patients following RTOG‐0813 dosing schemata via abdominal compression or 4D CT‐based treatment planning. This study indicates that treatment of lung SBRT on the Halcyon Linac is possible in a safe, feasible, and accurate manner and clinical experience to data conforms. For clinics equipped with only a Halcyon Linac, treatment of lung SBRT patients is possible and highly recommended.

## CONFLICT OF INTEREST

The author have no other relevant conflict of interest to disclose.

## AUTHOR'S CONTRIBUTIONS

DP conceived the project. JV, LC, JS, and DP collected and analyzed the data. DP, MB, MR, and MK provided clinical expertise and supervision of the paper. DP drafted the preliminary manuscript and all coauthors revised and approved the final manuscript.
